# LncR-133a Suppresses Myoblast Differentiation by Sponging miR-133a-3p to Activate the FGFR1/ERK1/2 Signaling Pathway in Goats

**DOI:** 10.3390/genes13050818

**Published:** 2022-05-03

**Authors:** Siyuan Zhan, Yang Zhang, Cuiting Yang, Dandan Li, Tao Zhong, Linjie Wang, Li Li, Hongping Zhang

**Affiliations:** 1 Key Laboratory of Livestock and Poultry Multi-Omics, Ministry of Agriculture and Rural Affairs, College of Animal and Technology, Sichuan Agricultural University, Chengdu 611130, China; siyuanzhan@sicau.edu.cn (S.Z.); zhongtao@sicau.edu.cn (T.Z.); wanglinjie@sicau.edu.cn (L.W.); 2 Farm Animal Genetic Resources Exploration and Innovation Key Laboratory of Sichuan Province, Sichuan Agricultural University, Chengdu 611130, China; yzhang0415@163.com (Y.Z.); fighting322@126.com (C.Y.); lidandan@sicau.edu.cn (D.L.); lily@sicau.edu.cn (L.L.)

**Keywords:** lncR-133a, muscle differentiation, miR-133a-3p, FGFR1, goat

## Abstract

Long noncoding RNAs (lncRNAs) are involved in a variety of biological processes and illnesses. While a considerable number of lncRNAs have been discovered in skeletal muscle to far, their role and underlying processes during myogenesis remain mostly unclear. In this study, we described a new functional lncRNA named lncR-133a. Gene overexpression and interference studies in goat skeletal muscle satellite cells (MuSCs) were used to establish its function. The molecular mechanism by which lncR-133a governs muscle differentiation was elucidated primarily using quantitative real-time PCR (qRT-PCR), Western blotting, dual-luciferase activity assays, RNA immunoprecipitation, biotin-labeled probe, and RNA fluorescence in situ hybridization analyses. LncR-133a was found to be substantially expressed in *longissimus thoracis et lumborum* muscle, and its expression levels changed during MuSC differentiation in goats. We validated that lncR-133a suppresses MuSC differentiation in vitro. Dual-luciferase reporter screening, Argonaute 2 (AGO2) RNA immunoprecipitation assays, biotin-labeled lncR-133a capture, and fluorescence in situ hybridization showed that lncR-133a interacted with miR-133a-3p. Additionally, miR-133a-3p facilitated MuSC differentiation, but lncR-133a reversed this effect. The luciferase reporter assay and functional analyses established that miR-133a-3p directly targets fibroblast growth factor receptor 1 (FGFR1). Moreover, lncR-133a directly reduced miR-133a-3p’s capacity to suppress FGFR1 expression, and positively regulated the phosphorylation of extracellular signal-regulated kinase 1/2 (ERK1/2). In summary, our results suggested that lncR-133a suppresses goat muscle differentiation by targeting miR-133a-3p and activating FGFR1/ERK1/2 signaling pathway.

## 1. Introduction

Skeletal muscle is the primary source of animal protein for human consumption, and its growth and development have a direct effect on meat quantity and quality [1]. Skeletal myogenesis is a well-regulated process that is regulated by a number of muscle-specific transcription factors, including the paired box family (Pax3 and Pax7), myogenic regulatory factor family (Myf5, Mrf4, MyoD, and MyoG), and myocyte enhancer factor 2 (MEF2) family [2,3,4,5,6]. However, muscle development processes, as well as the activities of novel regulatory elements and molecular mechanisms, must be investigated further. In recent years, a rising number of non-coding RNAs with the ability to regulate myogenesis have been found, including microRNAs, long non-coding RNAs, and circular RNAs. [7,8,9,10]. Thus, knowing the principles governing muscle growth and development is critical for genetically improving muscle quality.

Long noncoding RNAs (lncRNAs) are a family of transcripts longer than 200 nucleotides in length with limited capacity to code for proteins, low expression, and a low degree of conservation among species [11,12]. LncRNAs have a role in a variety of biological processes via epigenetic, transcriptional, and post-transcriptional regulation [9,13,14,15,16]. LncRNAs play a vital role in myogenesis. For instance, by competitively binding to miR-133 and miR-135, the muscle-specific linc-MD1 acts as a competing endogenous RNA (ceRNA) to upregulate the expression of myocyte enhancer factor 2C (MEF2C) and mastermind-like transcriptional coactivator 1 (MAML1), which activate muscle-specific gene expression and regulate myoblast differentiation in mouse and human [17]. Moreover, the RNA-binding protein human antigen R (HuR) stimulates the association between linc-MD1 and miR-133, which is critical for sustaining myogenic cells in the early differentiation stage [18]. The lncRNA lnc-mg stimulates myogenesis by acting as a ceRNA for microRNA-125b, hence regulating insulin-like growth factor 2 protein abundance [19]. LncMyoD is activated directly by MyoD and binds to IGF2-mRNA-binding protein 2 (IMP2), inhibiting the translation of proliferation genes such as N-Ras and c-Myc during myoblast differentiation [20]. SYNPO2 intron sense-overlapping lncRNA (SYISL) is strongly produced in skeletal muscle and directly inhibits muscle development by interacting with polycomb repressive complex 2 (PRC2) [21]. H19 functions as a scaffold for TDP43 recruitment to the MyoD promoter region, stimulating MyoD transcription and resulting in the differentiation of porcine satellite cells [22]. By binding to the protein interleukin enhancer-binding factor 3, the antisense lncRNA insulin-like growth factor 2 antisense transcript (IGF2 AS) enhances the proliferation and differentiation of bovine myoblasts. Additionally, IGF2 AS influenced the stability of maternal gene IGF2 and hence governed the development of bovine myoblasts [23]. Overall, lncRNAs regulate skeletal myogenesis in a variety of ways.

Understanding the mechanisms behind skeletal muscle development is essential to increase livestock meat production. Our previous study analysed the transcriptomes of fetal and postnatal goat skeletal muscle and discovered lncRNAs involved in muscle development [24]. In this study, lncR-133a is extensively expressed in *longissimus thoracis et lumborum* (LTL) muscle and is induced during the differentiation of skeletal muscle satellite cells (MuSCs). Furthermore, lncR-133a suppresses skeletal muscle differentiation by acting as a ceRNA and sponging miR-133a-3p, thereby augmenting the expression of fibroblast growth factor receptor 1 (FGFR1), which is a target gene of miR-133a-3p. This present study reports a novel pathway used by lncR-133a to regulate myogenesis and helps understand the regulation of muscle development by this lncRNA. Moreover, these data will provide the theoretical basis for the genetic improvement of goats.

## 2. Materials and Methods

### 2.1. Sample Preparation

Pregnant goats (aged 2–3 years) were used in this study. The goats were kept in a free-stall barn and were fed a standard diet (forage to concentrate ratio, 65:35) twice daily (06:30–08:30 and 16:00–18:00) with free access to water. Twelve fetuses were removed through humane caesarean section at 45, 60, 75, and 105 days of gestation (E45, E60, E75, and E105). In addition, three female goats were sacrificed humanely on the third day after birth (B3). *Longissimus thoracis et lumborum* (LTL) muscle samples were obtained from these five developmental stages, and heart, liver, spleen, lung, and kidney samples were collected at 60 days of gestation. All samples were frozen in liquid nitrogen for RNA extraction.

### 2.2. RNA Extraction and Quantitative Real-Time PCR

Total RNA was extracted from tissues or cells using TRIzol Reagent (Invitrogen, Carlsbad, CA, USA) according to the manufacturer’s instructions. RNA quality was assessed by electrophoresis on a 1.5% agarose gel, and RNA concentration was measured using a NanoDrop 2000 Spectrophotometer (Thermo-Fisher Scientific, Waltham, MA, USA). RNA (~1 μg) was reverse-transcribed into cDNA using the PrimeScript^TM^ RT Reagent Kit with gDNA Eraser (Takara, Dalian, China) (for mRNA and lncRNA detection) and the Mir-X^TM^ miRNA First-Strand Synthesis Kit (Takara, Dalian, China) (for miRNA assays). Quantitative real-time PCR (qRT-PCR) was performed using the SYBR Premix Ex Taq^TM^ II or Mir-X^TM^ miRNA qRT-PCR SYBR^®^ Kit (Takara, Dalian, China). PCR was performed in a 10 μL volume containing 5 μL of SYBR, 0.4 μL of each primer (10 μM), 0.8 μL of normalized template cDNA (~2 μg/μL), and 3.4 μL of sterile water. Amplification conditions were 95 °C for 2 min followed by 40 cycles at 95 °C for 10 s and annealing temperature for 30 s, as shown in Supplementary Table S1. Melting curve analysis was performed from 65 °C to 95 °C with an incremental increase of 0.5 °C per second. Each experiment was performed with three biological replicates and repeated independently three times. Relative expression levels were calculated using the 2^−ΔΔCt^ method [25]. The genes ACTB and U6 (for miRNA) served as internal controls. Supplementary Table S1 contains a list of the primers used in this work. Primers were designed using Primer-BLAST (http://www.ncbi.nlm.nih.gov/tools/primer-blast/, accessed on 8 May 2021), and were synthesized by the Sangon Biotechnology Company (Shanghai, China).

### 2.3. Vector Construction, RNA Oligonucleotides, and Transfection

The construction of overexpression plasmids involved the synthesis of the full-length lncR-133a by TsingKe (Beijing, China) followed by cloning into the pEGFP-N1 vector (Promega, Madison, WI, USA) using the restriction enzymes *XhoI* and *BamHI* to obtain the expression plasmid pEGFP-lncR-133a. The primers used for plasmid construction are listed in Supplementary Table S1. Three siRNAs independently targeting lncR-133a (si-lncR-133a: CCATCTACTAGCTGAATAA, CCTGATGTGTTTCAGTATT, CCGTCTGGTCAGTCTCTAA) were designed and synthesized by RiboBio (Guangzhou, China). Non-specific siRNA sequences were used as negative controls, and a mixture of three siRNAs was used for lncR-133a interference experiments. MiR-133a-3p mimics, negative control mimics, a miR-133a-3p inhibitor, and a negative control inhibitor were designed and synthesized by RiboBio (Guangzhou, China). Goat skeletal muscle satellite cells (MuSCs) were transfected with plasmid DNA using Lipofectamine 2000 (Invitrogen, Carlsbad, CA, USA) according to the manufacturer’s specifications. The final concentration of plasmids and RNA oligos used in transfections or co-transfections was 50 nM.

### 2.4. Cell Culture and Treatment

MuSCs from the LTL muscle of neonatal goats were isolated and cultured as previously described [26]. MuSCs were seeded in 6-well (~2 × 10^4^ cells per well) or 12-well (~1 × 10^4^ cells per well) plates containing growth medium (GM) (Dulbecco’s Modified Eagle Medium [DMEM] supplemented with 15% FBS) and cultured at 37 °C in a 5% CO_2_ atmosphere. GM was replaced with differentiation medium (DM) containing 2% horse serum (98% DMEM high glucose + 2% HS; Gibco) to induce myoblast differentiation when MuSCs reached 80–90% confluence. The medium was changed every 2 days.

To assess gain or loss of function, GM was replaced with DMEM containing 15% FBS when cells reached 80–90% confluence, followed by Lipofectamine 2000 (Invitrogen, Carlsbad, CA, USA) transfection. After 6 h of transfection, GM was replaced with DM. After 48 h, the transfected cells were collected for qRT-PCR and Western blot assays.

### 2.5. Immunofluorescence Analyses

MuSCs (seeded at ~2 × 10^4^ cells per dish) were transfected in 3.5-cm Petri dishes and cultured in DM. After 96 h of transfection, the cells were fixed in 4% paraformaldehyde for 15 min at room temperature and washed three times with 1 mL of PBS. The cells were permeabilized with 1 mL of 0.5% Triton X-100 at 4 °C for and washed with PBS three times. Samples were blocked with 1 mL of 2% bovine serum albumin for 30 min at 37 °C and incubated overnight with a monoclonal antibody to myosin heavy chain (MyHC) (1:150; Abcam, Cat. No. ab51263, Cambridge Science Park, Cambridge, UK) at 4 °C with gentle shaking. The cells were washed three times with PBS (5 min each) and incubated with Cy3-conjugated IgG (H + L) (1:150, Cell Signaling Technology, Cat. No. 4410, Beverly, MA, USA) at 37 °C for 2 h. The cells were rinsedthree times with PBS and stained with 0.05 μg/mL DAPI (4′,6′-diamidino-2-phenylindole; Invitrogen) at 37 °C for 10 min in a humidified dark chamber. The cells were washed three times with 1 mL of PBS and imaged on a fluorescent microscope (Leica, Wetzlar, Germany). The differentiation index was calculated as the percentage of nuclei in MyHC-positive cells, and the fusion index was calculated as the percentage of nuclei in fused myotubes with two or more nuclei. At least three samples were examined separately for each treatment, and five areas per sample were randomly selected.

### 2.6. Western Blot Analysis

The antibodies used were MyoG (ab124800, Abcam), MyoD (ab133627, Abcam), p-ERK1/2 (9101, Cell Signaling Technology), GAPDH (ab8245, Abcam), and horseradish peroxidase (HRP)-conjugated anti-rabbit IgG (A0208; Beyotime). Total protein from cultured MuSCs was extracted using the Total Protein Extraction Kit (Beyotime) and quantified using the BCA Protein Quantitation Kit (Beyotime) according to the manufacturer’s instructions. Briefly, proteins (~20 μg per sample) were separated by 10% sodium dodecyl sulphate-polyacrylamide gel electrophoresis, transferred to a PVDF membrane (Millipore, Bedford, MA, USA), and blocked with blocking buffer (Beyotime) for 1 h at room temperature. The membranes were sequentially incubated with primary anti-mouse MyoG (1:1000), MyoD (1:1000) and p-ERK1/2 (1:1000), washed three times with PBST (0.1% Tween 20 in PBS), and incubated with the secondary antibody conjugated with HRP (1:4000) for 90 min. The membranes were washed three with PBST. Immunoreactive bands were visualized using an enhanced chemiluminescence detection system (BeyoECL Plus, Beyotime) and the ChemiDoc XRS^+^ system (Bio-Rad, Hercules, CA, USA). GAPDH (1:2000) served as a loading control.

### 2.7. Luciferase Activity Assay

Wild-type and mutant lncR-133a fragments and the 3′-untranslated region (UTR) of the FGFR1 gene were amplified and subcloned into the psiCHECK-2 vector (Promega, Madison, WI, USA) using *XhoI* and *NotI* restriction enzymes (Takara, Dalian, China). LncR-133a and FGFR1 mutants were generated by changing the binding site from GACCAA to GAATGC and from GGACCAA to GGTGAGC, respectively. The primers used for plasmid construction are listed in Supplementary Table S1. All constructs were confirmed by Sanger sequencing. For the luciferase reporter assays, MuSCs (~1 × 10^4^ cells per well) were seeded in 24-well plates containing GM under experimental conditions similar to those for MuSCs, followed by transfection when the cells reached 80–90% confluence. GM was replaced with DM after 6 h of transfection. HEK293T cells were cultured in 48-well plates until they reached 70–80% confluence and were transfected with the plasmids. Transfected cells were harvested and lysed using cell lysis buffer (NEB, Ipswich, MA, USA), and 48 h after differentiation, luciferase activity was determined using a dual-luciferase reporter assay kit (Promega, Madison, WI, USA) according to the manufacturer’s instructions. Firefly luciferase activity was normalized to Renilla luciferase activity.

### 2.8. Nuclear and Cytoplasmic RNA Fractionation

Nuclear/cytoplasmic fractionation of MuSCs was performed using a cytoplasmic/nuclear RNA purification kit (Norgen Biotek, Thorold, ON, Canada) according to the manufacturer’s instructions. Briefly, MuSCs (seeded at ~2 × 10^7^ cells per dish) cultured on a 10-cm culture dish were washed twice using cold PBS after 3 days of differentiation and were lysed in a hypotonic buffer (10 mM Tris pH 8.0, 1 mM EDTA). To separate nuclei and debris, cells were centrifuged at 500× *g* for 5 min. Total RNA was extracted separately from the supernatant (cytosolic fraction) and the pellet (nuclei) using RNAiso Plus reagent (TaKaRa, Dalian, China) and reverse-transcribed into cDNA using the PrimeScript^TM^ RT Reagent Kit and gDNA Eraser (Takara, Dalian, China). RNA was quantified by qRT-PCR. 18S ribosomal RNA (18S rRNA) and U6 were used as cytoplasmic and nuclear controls, respectively. All procedures were performed at 4 °C under RNase-free conditions.

### 2.9. RNA-Binding Protein Immunoprecipitation (RIP) Assay

RIP assays were performed using the Magna RIP™ RNA-Binding Protein Immunoprecipitation Kit (Millipore, Billerica, MA, USA) following the manufacturer’s protocol. MuSCs (~2 × 10^7^ cells) grown for 72 h were harvested and lysed in RIP lysis buffer. The cells were divided into two equal parts and incubated overnight at 4 °C with magnetic beads conjugated with an antibody IgG (Millipore, Billerica, MA, USA) or AGO2 (Abcam, Cambridge Science Park, Cambridge, UK). LncR-133a and miR-133a-3p in immunoprecipitated RNA were quantified by qRT-PCR.

### 2.10. Biotin-Labeled lncR-133a Capture

MuSCs were transfected with a biotinylated lncR-133a probe and harvested at 48 h after transfection, as previously described [19]. The cells were lysed on ice for 30 min in 250 μL of cell lysis buffer (10 mM KCl, 1.5 mM MgCl_2_, 10 mM Tris-HCl pH 7.5, 5 mM dithiothreitol). The supernatant was centrifuged for 5 min at 12,000× *g*, and the beads were washed five times with solution A (0.1 M NaOH and 0.05 M NaCl) and thrice with 0.1 M NaCl. The beads were blocked with 1 mg/mL bovine serum albumin (Roche, Basel, Switzerland) and 1 mg/mL yeast transfer RNA (Thermo Fisher Scientific, Waltham, MA, USA) overnight. Subsequently, 500 μL NaCl (1 M) and 30 μL beads were added to the supernatant. The beads were washed five times in washing buffer (5 mM Tris-HCl pH 7.5, 0.5 mM EDTA, 1 M NaCl) and mixed with the lysate for 4 h at 4 °C. The RNA complexes were bound to the beads were eluted and extracted for qRT-PCR. PCR assays were performed as described in the subheading *RNA extraction and quantitative real-time PCR*.

### 2.11. RNA Dual-Labeled Fluorescence In Situ Hybridization (FISH)

The fluorescence oligonucleotide probes complementary to lncR-133a (5′-GACATCAGTGGCGAAGGTGGAGAATGGACAGCA-3′, labeled with Cy5) and miR-133a-3p (5′-ACAGCTGGTTGAAGGGGACCAAA-3′, labeled with Cy3) were synthesized by Servicebio (Wuhan, China) to detect the interaction between lncR-133a and miR-133a-3p. MuSCs cultured for 3 days were fixed in 4% DEPC-treated paraformaldehyde for 20 min at room temperature. Paraformaldehyde-fixed cells were washed three times (5 min each time) using PBS and pre-hybridized for 1 h in wash buffer (2 × SSC, 10% formamide) at room temperature. The cells were hybridized using 8 ng/μL probe in 50 μL of buffer (2 × SSC, 10% formamide, 10% dextran sulfate) for 16 h at 37 °C in the dark. The cells were rinsed using 2 × SSC (Servicebio) and washed with PBS for 5 min. Cells were stained using 0.05 μg/mL DAPI (Invitrogen) for 30 min at 37 °C and imaged on a Nikon Eclipse TiSR microscope equipped with a Nikon DS-U3 digital camera. Images were analyzed using CaseViewer software.

### 2.12. Statistics Analysis

Data are means ±standard error of the mean of at least three biological replicates. Statistical significance was analyzed using the Student’s *t*-test for comparisons of two groups, and one-way analysis of variance with Tukey’s post-hoc test for multiple groups using SAS software version 9.0 (SAS, Cary, NC, USA). The significance level was set at *p* < 0.05.

## 3. Results

### 3.1. Coding Potential and Expression Profile of lncR-133a

**Figure 1:**
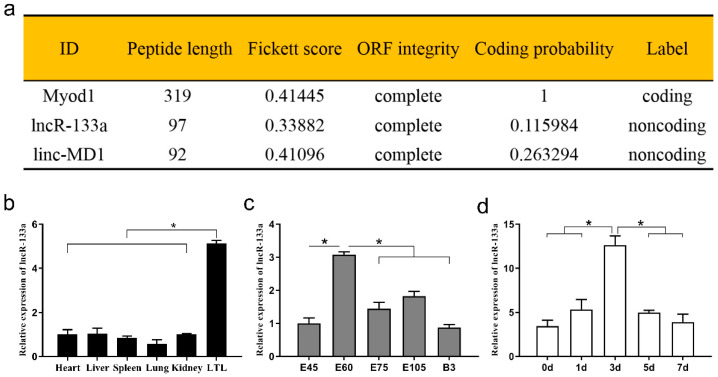
Coding potential and expression profile of lncR-133a. (**a**) Bioinformatics analysis of the coding potential of lncR-133a, and Myod1 and linc-MD1 served as controls. (**b**) qRT-PCR analysis of lncR-133a expression in heart, liver, spleen, lung, kidney, and longissimus thoracis et lumborum (LTL) muscle on day 60 of gestation. (**c**) The expression pattern of lncR-133a in the developmental LTL muscle. (**d**) The expression levels of lncR-133a during goat MuSC differentiation. Data are means ± standard error of the mean of at least three biological replicates, * *p* < 0.05.

Our previous study found that several lncRNAs, including the 1581-nucleotide lncR-133a (TCONS_00067594), were differentially expressed in fetal and neonatal muscle tissue of goats [24] and that lncR-133a was located on chromosome 16 (Supplementary Figure S1). The present study evaluated the coding potential of lncR-133a using CPC2 online software (http://cpc2.cbi.pku.edu.cn/, accessed on 12 March 2021) and revealed that the coding potential of lncR-133a was weaker than that of the controls Myod1 and linc-MD1 (Figure 1a). Moreover, all goat tissues examined (heart, liver, spleen, lung, kidney, and LTL) expressed lncR-133a and expression was highest in the LTL muscle (*p* < 0.05) (Figure 1b). In LTL tissue, lncR-133a expression peaked on day 60 of gestation (*p* < 0.05) (Figure 1c). The expression level of four marker genes was also measured in said tissues (Supplementary Figure S2a,b), and the expression levels of myogenesis markers (Pax7, MyoD, MyoG, MyHC) during MuSC differentiation were quantified (Supplementary Figure S2c). The expression pattern of these markers during MuSC differentiation agreed with a previous study [27] and demonstrated that lncR-133a expression could be quantified accurately. The expression levels of lncR-133a during goat MuSC differentiation were also measured in vitro (growth medium [GM] and differentiation medium [DM] for 1, 3, 5, and 7 d). The results indicated that lncR-133a was upregulated in the early stage of differentiation and reached its peak in MuSCs differentiated for 3 d (*p* < 0.05) followed by a gradual decrease (Figure 1d).

### 3.2. LncR-133a Suppresses the Differentiation of Goat MuSCs

**Figure 2:**
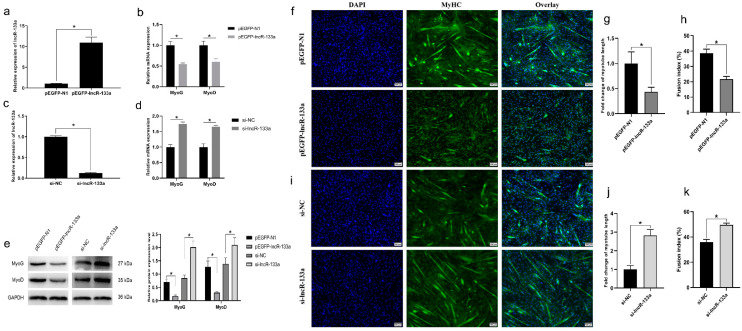
LncR-133a suppresses the differentiation of goat MuSCs. (**a**) The expression levels of lncR-133a were determined in MuSCs transfected with pEGFP-N1 or pEGFP-lncR-133a. (**b**) Muscle differentiation marker genes (MyoG and MyoD) expression levels were determined in MuSCs transfected with pEGFP-N1 or pEGFP-lncR-133a. (**c**) The expression levels of lncR-133a were measured in MuSCs transfected with si-NC or si-lncR-133a. (**d**) The expression levels of muscle differentiation marker genes (MyoG and MyoD) were determined in MuSCs transfected with si-NC or si-lncR-133a. (**e**) MyoG and MyoD protein levels were determined in MuSCs transfected with pEGFP-N1 or pEGFP-lncR-133a, si-NC or si-lncR-133a. Image Pro Plus software (version 6.0, Media Cybernetics) was used to calculate the quantification of protein expression. (**f**) MyHC-stained cells were transfected with pEGFP-N1 or pEGFP-lncR-133a and then cultured in differentiation medium (DM) for 4 days. DAPI stainingwas used to visualize the nuclei of cells (blue). The length (**g**) and fusion index (**h**) of myotubes were determined. Scale bars = 100 μm. (**i**) Immunofluorescence assay for MyHC-stained cells transfected with si-NC or si-lncR-133a then cultured in DM for 4 days. Cell nuclei were visualized with DAPI (blue). Quantified myotube length (**j**) and fusion index (**k**) Scale bars = 100 μm. Data are means ± standard error of the mean of at least three biological replicates, * *p* < 0.05.

To study the role of lncR-133a in the differentiation of goat MuSCs, we constructed the lncR-133a overexpression vector pEGFP-lncR-133a and synthesized the siRNA si-lncR-133a to suppress lncR-133a. qRT-PCR results showed that lncR-133a was successfully overexpressed in this vector and inhibited by si-lncR-133a in MuSCs (*p* < 0.05; Figure 2a,c). Changes in mRNA and protein expression of muscle differentiation marker genes (MyoG and MyoD) and myotube formation as a function of lncR-133a levels were assessed. The results showed that lncR-133a overexpression significantly decreased the mRNA and protein abundance of MyoG and MyoD (*p* < 0.05; Figure 2b,e), whereas lncR-133a knockdown had the opposite effect (*p* < 0.05; Figure 2d,e). To confirm that lncR-133a inhibits MuSC differentiation, immunofluorescence analysis was performed to detect myosin heavy chain positive (MyHC^+^) cells transfected with pEGFP-N1 or pEGFP-lncR-133a and cultured in DM for 4 days. Compared with the negative control (NC), lncR-133a overexpression significantly downregulated MyHC expression, whereas lncR-133a knockdown had the opposite effect (Figure 2f,i). Furthermore, lncR-133a overexpression strongly decreased myotube length (Figure 2g) and the fusion index (Figure 2h), whereas lncR-133a knockdown had the opposite effect (Figure 2j,k). These results indicate that lncR-133a suppresses the differentiation of goat MuSCs.

### 3.3. LncR-133a Acts as a Molecular Sponge for miR-133a-3p

**Figure 3:**
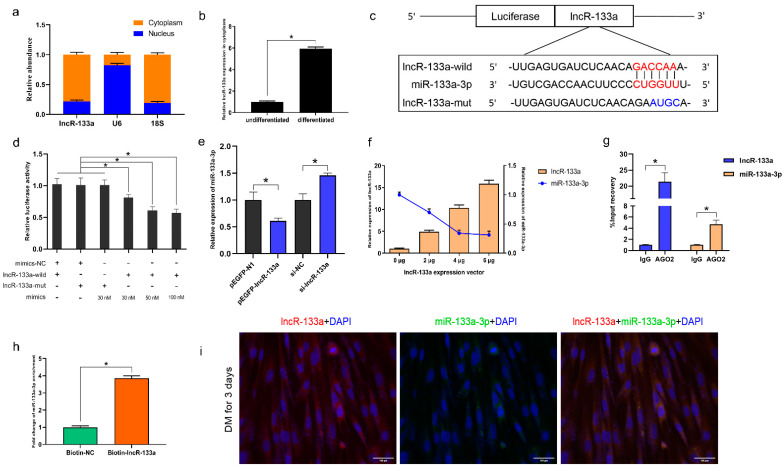
LncR-133a acts as a molecular sponge for miR-133a-3p. (**a**) The relative abundance of lncR-133a in the cytoplasm and nuclei of MuSCs differentiated for 3 days. 18S rRNA and U6 served as cytoplasmic and nuclear controls, respectively. (**b**) qRT-PCR analysis of lncR-133a level in the cytoplasm of undifferentiated MuSCs and MuSCs differentiated for 3 days. (**c**) Schematic diagram of a luciferase reporter containing lncR-133a wild type or mutant type of miR-133a-3p targeting site. The seed sequence for miR-133a-3p is displayed in red, whereas the mutant sequence for lncR-133a is indicated i in blue. (**d**) Luciferase assays demonstrated that miR-133a-3p inhibits the activity of lncR-133a. (**e**) qRT-PCR analysis of miR-133a-3p expression in MuSCs transfected with pEGFP-N1, pEGFP-lncR-133a, si-NC, and si-lncR-133a, respectively. (**f**) qRT-PCR evaluation of the expression levels of lncR-133a and miR-133a-3p in MuSCs transfected with increasing concentrations of lncR-133a expression vector. (**g**) RIP assay was carried out using AGO2 antibody, with IgG as the negative control. (**h**) The expression of miR-133a-3p was measured after streptavidin capture in MuSCs (3 d) transfected with biotin-NC and biotin-lncR-133a. (**i**) Co-localization of lncR-133a and miR-133a-3p in MuSCs. RNA Dual-labeled Fluorescence in Situ Hybridization (FISH) assay was performed on MuSCs cultured for 3 d, using fluorescent oligonucleotide probes complementary to lncR-133a (red) and miR-133a-3p (green). Cell nuclei were visualized with DAPI staining (blue), Scale bars = 100 μm. Data are means ± standard error of the mean of at least three biological replicates, * *p* < 0.05.

LncR-133a is found in the cytoplasm and nucleus (Figure 3a), and its expression in the cytoplasm increases significantly in differentiated myoblasts (Figure 3b). We speculate that lncR-133a may function as a molecular sponge of miRNA in myogenic differentiation. To test this hypothesis, miRNAs targets were predicted using TargetScan [28] and RNAhybrid software [29], and the results showed that lncR-133a contained a potential binding site for miR-133a-3p (Figure 3c). A dual-luciferase reporter system, in which the wild-type sequence or a mutated sequence of lncR-133a was bound to the 3′ end of the firefly luciferase gene, was used to confirm whether lncR-133a interacted with miR-133a-3p (Figure 3c). The results revealed that miR-133a-3p suppressed the luciferase activity of the wild-type but not the mutated lncR-133a sequence (Figure 3d), demonstrating that miR-133a-3p binds to lncR-133a during myoblast differentiation by complementary base pairing. In addition, lncR-133a overexpression downregulated miR-133a-3p in a dose-dependent manner, whereas lncR-133a knockdown had the opposite effect (Figure 3e,f). Argonaute 2 (AGO2) immunoprecipitation and biotin-labelled lncR-133a capture followed by real-time PCR confirmed the interaction between miR-133a-3p and lncR-133a (Figure 3g,h). Furthermore, the results of RNA fluorescence in situ hybridization (FISH) assays using fluorescence oligonucleotide probes for lncR-133a and miR-133a-3p showed that that lncR-133a and miR-133a-3p completely overlapped in the cytoplasm on day 3 of MuSC differentiation (Figure 3i). These results indicate that lncR-133a regulates miR-133a-3p during myoblast differentiation by functioning as a molecular sponge.

### 3.4. MiR-133a-3p Promotes MuSC Differentiation

**Figure 4:**
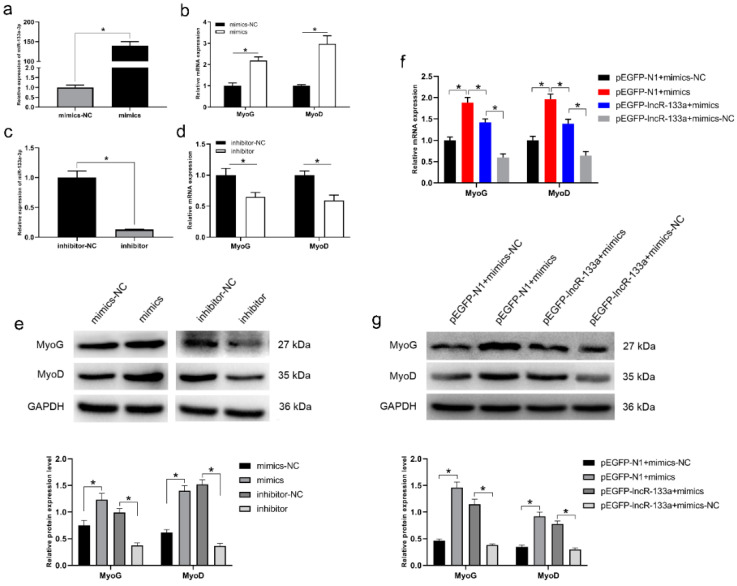
MiR-133a-3p promotes MuSC differentiation. (**a**) The expression level of miR-133a-3p was measured in MuSCs transfected with miR-133a-3p mimics (mimics) or mimics negative control (mimics-NC). (**b**) The expression levels of MyoG and MyoD were determined in MuSCs transfected with miR-133a-3p mimics or mimics-NC. (**c**) The expression level of miR-133a-3p was measured in MuSCs transfected with miR-133a-3p inhibitor (inhibitor) or inhibitor negative control (inhibitor-NC). (**d**) The expression levels of MyoG and MyoD were determined in MuSCs transfected with miR-133a-3p inhibitor or inhibitor-NC. (**e**) The protein levels of MyoG and MyoD were measured in MuSCs transfected with miR-133a-3p mimics or mimics-NC, miR-133a-3p inhibitor or inhibitor-NC. (**f**) The expression levels MyoG and MyoD were determined after treatment with miR-133a-3p mimics and/or pEGFP-lncR-133a. (**g**) The protein levels of MyoG and MyoD in MuSCs transfected with miR-133a-3p mimics and/or pEGFP-lncR-133a. Data are means ± standard error of the mean of at least three biological replicates, * *p* < 0.05.

MiR-133a-3p expression was evaluated using a miR-133a-3p mimic and inhibitor. The miR-133a-3p mimic significantly increased miR-133a-3p levels in MuSCs, whereas the miR-133a-3p inhibitor significantly decreased miR-133a-3p levels (*p* < 0.05; Figure 4a,c). In addition, MyoG and MyoD expression was also examined. The mRNA and protein abundance of MyoG and MyoD was increased by the miR-133a-3p mimic and decreased by the miR-133a-3p inhibitor (*p* < 0.05; Figure 4b,d,e). These results indicate that miR-133a-3p promotes MuSC differentiation.

### 3.5. LncR-133a Reverse the Effect of miR-133a-3p on MuSC Differentiation

Our results suggest that lncR-133a binds to and inhibits miR-133a-3p during MuSC differentiation. Therefore, rescue experiments were performed to assess whether the effect of miR-133a-3p on MuSC differentiation was blocked by lncR-133a overexpression. The qRT-PCR and Western blot analysis results indicated that lncR-133a overexpression blocked the ability of miR-133a-3p to increase the mRNA and protein levels of MyoG and MyoD (*p* < 0.05; Figure 4f,g).

### 3.6. FGFR1 Is a Target Gene of miR-133a-3p and Inhibits Myoblast Differentiation

**Figure 5:**
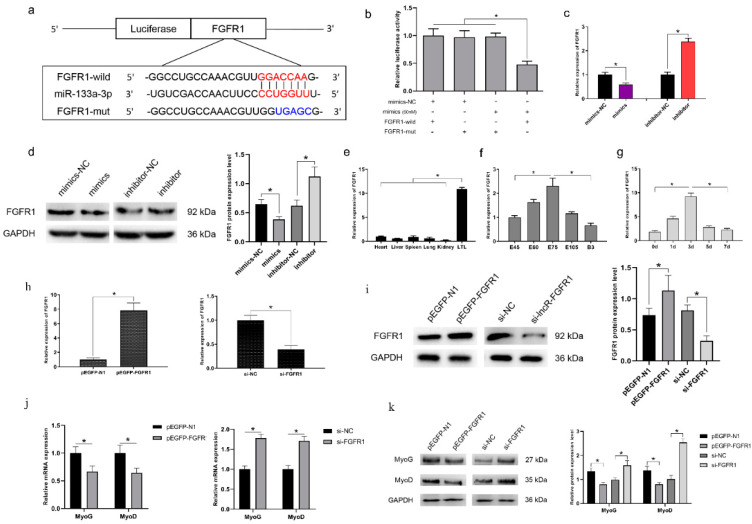
FGFR1 is a target gene of miR-133a-3p and inhibits myoblast differentiation. (**a**) Vectors containing the FGFR1 wild-type (FGFR1-wild) and mutant (FGFR1-mut) sequences were constructed. The miR-133a-3p seed sequence and the FGFR1 mutant sequence are indicated in red and blue, respectively. (**b**) Luciferase assays revealed the suppressive effect of miR-133a-3p on the activity of FGFR1. (**c**) The expression level of FGFR1 in MuSCs transfected with miR-133a-3p mimics or inhibitor. (**d**) The protein expression of FGFR1 in MuSCs transfected with miR-133a-3p mimics or inhibitor. (**e**) The expression pattern of FGFR1 in goat tissues. (**f**) The expression pattern of FGFR1 in the developmental LTL muscle. (**g**) qRT-PCR analysis of FGFR1 expression in MuSCs during 7 days of differentiation. (**h**) qRT-PCR analysis of FGFR1 expression in MuSCs transfected with pEGFP-FGFR1 or si-FGFR1 after 48 h in DM. (**i**) The protein expression of FGFR1 in MuSCs transfected with pEGFP-FGFR1 or si-FGFR1 after 48 h in DM. (**j**) qRT-PCR analysis of mRNA expression of MyoG and MyoD in MuSCs transfected with pEGFP-FGFR1 or si-FGFR1 then cultured in DM for 48 h. (**k**) Western blot analysis of MyoG and MyoD protein expression in MuSCs transfected with pEGFP-N1, pEGFP-FGFR1, si-NC, and si-FGFR1, respectively. Image Pro Plus software (version 6.0, Media Cybernetics) was used to compute the quantification of protein expression. Data are means ± standard error of the mean of at least three biological replicates, * *p* < 0.05.

The genes targeted by miR-133a-3p were identified using TargetScan (http://www.targetscan.org, accessed on 21 June 2021). The results revealed that FGFR1 was the strongest candidate because of its well-established role in C2C12 myoblast differentiation [30]. In addition, RNAhybrid software predicted that the FGFR1 3′ UTR had a binding site for miR-133a-3p (Figure 5a). A luciferase reporter assay was conducted to confirm whether miR-133a-3p directly targeted FGFR1, and the results showed that miR-133a-3p targeted the wild-type 3′ UTR but not the mutant UTR (Figure 5b). In addition, FGFR1 mRNA and protein expression was downregulated by miR-133a-3p overexpression and upregulated by miR-133a-3p knockdown (Figure 5c,d), suggesting that miR-133a-3p inhibits FGFR1 expression during MuSC differentiation. Furthermore, we evaluated the expression pattern of FGFR1 in goat tissues and cultured MuSCs using qRT-PCR. FGFR1 expression was relatively high in the LTL muscle (*p* < 0.05) (Figure 5e) and peaked on day 75 of gestation (Figure 5f). In cultured MuSCs, FGFR1 expression peaked on day 3 of differentiation (Figure 5g).

To study the effect of FGFR1 on myoblast differentiation, we constructed the overexpression vector pEGFP-FGFR1 and synthesized a siRNA targeting FGFR1. The results showed that FGFR1 was successfully overexpressed in this vector and was inhibited by si-FGFR1 in MuSCs (Figure 5h,i). Moreover, FGFR1 overexpression significantly decreased the mRNA and protein expression of MyoG and MyoD (*p* < 0.05), whereas FGFR1 knockdown had the opposite effect (*p* < 0.05, Figure 5j,k). These data suggest that FGFR1 inhibits myoblast differentiation.

### 3.7. LncR-133a Increases FGFR1 Expression via miR-133a-3p and Promotes the Phosphorylation of ERK1/2

**Figure 6:**
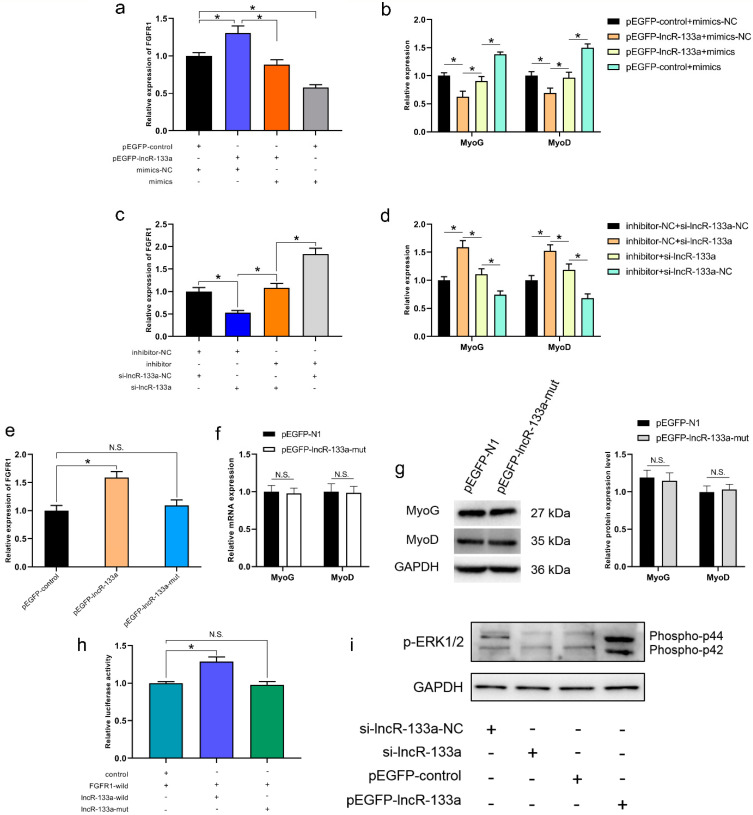
LncR-133a regulates FGFR1 expression and MuSCs differentiation via miR-133a-3p and promotes the phosphorylation of ERK1/2. (**a**) The expression of FGFR1 was determined after treatment with pEGFP-lncR-133a and/or miR-133a-3p mimics. (**b**) The expression of MyoG and MyoD were detected after treatment with pEGFP-lncR-133a and/or miR-133a-3p mimics. (**c**) The expression of FGFR1 was measured after treatment with miR-133a-3p inhibitor and/or si-lncR-133a. (**d**) The expression of MyoG and MyoD were detected after treatment with si-lncR-133a and/or miR-133a-3p inhibitor. (**e**) The expression level of FGFR1 in MuSCs transfected with pEGFP-lncR-133a or pEGFP-lncR-133a-mut. (**f**) The mRNA expression of MyoG and MyoD in MuSCs transfected with pEGFP-N1 or pEGFP-lncR-133a-mut. (**g**) The protein expression of MyoG and MyoD were determined in MuSCs transfected with pEGFP-N1 or pEGFP-lncR-133a-mut. Image Pro Plus software (version 6.0, Media Cybernetics) was used to compute the quantification of protein expression. (**h**) To test lncR-133a’s competitive endogenous RNA activity, FGFR1-wild was co-transfected into HEK293T cells with lncR-133a-wild or lncR-133a-mut. (**i**) LncR-133a promotes the phosphorylation of ERK1/2 during myoblast differentiation. In addition, pEGFP-lncR-133a, si-lncR-133a and Duplex NC were transfected into MuSCs. Next, cells were switched into the differentiation medium approximately 6 h after transfection. The total protein from transfected cells were isolated after culturing in the differentiation medium for 48 h. Data are means ± standard error of the mean of at least three biological replicates, * *p* < 0.05.

Due to the fact that lncR-133a and FGFR1 share miRNA-response domains, we evaluated whether lncR-133a might directly bind to miR-133a-3p and regulate FGFR1 expression. The results showed that lncR-133a overexpression increased FGFR1 expression and decreased MyoG and MyoD level, and miR-133a-3p attenuated this effect (Figure 6a,b). LncR-133a knockdown decreased FGFR1 expression and increased MyoG and MyoD expression, whereas the miR-133a-3p inhibitor abrogated this effect (Figure 6c,d), demonstrating that miR-133a-3p modulates the effect of lncR-133a on FGFR1 and myoblast differentiation. Moreover, lncR-133a lacking miR-133a-3p binding sites did not significantly affect FGFR1 expression and the mRNA and protein abundance of MyoG and MyoD (Figure 6e–g), indicating that the observed effect was controlled by miR-133a-3p.

To confirm whether lncR-133a affected FGFR1 through miR-133a-3p, the effect of lncR-133a was assessed using a luciferase reporter system. lncR-133a induced Renilla luciferase expression in the wild-type 3′ UTR of FGFR1 but not in the mutant UTR (Figure 6h). A previous study suggested that miR-133 promoted myoblast differentiation by repressing the phosphorylation of extracellular signal-regulated kinase 1/2 (ERK1/2) through targeting FGFR1 [30]. To explore the potential connection between lncR-133a and ERK1/2 phosphorylation, we evaluated whether lncR-133a silencing and overexpression affected the phosphorylation of ERK1/2. The results indicated that pEGFP-lncR-133a promoted ERK1/2 phosphorylation, whereas si-lncR-133a had the opposite effect (Figure 6i).

## 4. Discussion

Skeletal myogenesis is tightly controlled by a complex network, and accumulating evidence suggests that lncRNAs play an important role in this process [9,31]. A few lncRNAs are known to play a role in goat skeletal myogenesis. This study found that lncR-133a was significantly expressed in skeletal muscle and suppressed the differentiation of goat skeletal muscle satellite cells by sponging miR-133a-3p, consequently increasing FGFR1 expression. Several lncRNAs have been identified in the skeletal muscle of pig [32,33,34], cattle [35,36,37], sheep [38,39,40], chicken [41,42], and goats [43]. Moreover, we previously identified lncRNAs in fetal and postnatal goat skeletal muscle using RNA-seq technology [24]. These data provide a basis for understanding the role of lncRNAs in goat muscle formation.

This study found that lncR-133a was highly expressed in the LTL muscle of embryonic goats, and expression changed during muscle development and MuSC differentiation, suggesting that lncR-133a might be involved in the regulation of muscle development. One of the characteristics of lncRNAs is spatio-temporal changes in expression patterns [12,42,43], consistent with our results. The effect of lncR-133a overexpression and knockdown on MuSC differentiation was assessed in vitro. LncR-133a overexpression suppressed myogenic differentiation, whereas lncR-133a silencing had the opposite effect, consistent with the effects of lncRNA 2310043L19Rik [44] and AK017368 [45]. For instance, lncRNA 2310043L19Rik overexpression inhibited myoblast differentiation but promoted cell proliferation, whereas lncRNA 2310043L19Rik silencing had the opposite effect in vitro [44]. Similarly, lncRNA AK017368 inhibited myoblast differentiation, and AK017368 gain/loss-of-function had the opposite effect [45].

Bioinformatics analysis of the lncR-133a sequence was done to elucidate the molecular mechanisms by which lncR-133a regulates muscle differentiation, and the results showed this RNA contains a potential binding site for miR-133a-3p. Dual-luciferase reporter assays confirmed that lncR-133a is a direct target of miR-133a-3p. The regulatory action of lncRNAs is dictated by their subcellular localization. In this respect, many cytoplasm-located lncRNAs act as ceRNAs by sponging miRNAs, increasing the expression of target genes. For instance, muscle-specific linc-MD1 reduces the inhibition of MAML1 and MEF2C and induces skeletal muscle differentiation by sponging miR-133 and miR-135, respectively [17]. Therefore, RNA-FISH and subcellular localization analysis of lncR-133a was done in this study, and the results indicated that lncR-133a was localized to the cytoplasm, suggesting that lncR-133a might affect myogenesis by acting as a ceRNA and sponging miR-133a-3p. AGO2-RIP and biotin-labeled lncR-133a capture experiments confirmed that lncR-133a sponged miR-133a-3p during MuSC differentiation. In addition, miR-133a-3p expression was inhibited by lncR-133a, suggesting that lncR-133a interacted with miR-133a-3p.

Several studies have reported that miR-133, one of the most abundant myogenic microRNAs, regulates multiple targets involved in skeletal muscle development. The transcription factor serum response factor, which is required for muscle proliferation and differentiation, was the first target discovered [46]. In addition, miR-133 participates in myoblast proliferation and differentiation by negatively modulating IGF-1R/PI3K/Akt signaling through the inhibition of IGF-1R [47]. Another study has reported that miR-133 inhibits myoblast proliferation and promotes myoblast differentiation by repressing the ERK1/2 pathway via FGFR1 and PP2AC [30]. Furthermore, gga-miR-133a-3p inhibits chicken myoblast proliferation and promotes myoblast differentiation by targeting PRRX1 [48]. In this study, miR-133a-3p significantly increased muscle differentiation-related gene expression, whereas lncR-133a had the opposite effect. Furthermore, lncR-133a reduced the stimulatory effect of miR-133a-3p on MuSC differentiation, suggesting that lncR-133a sponged miR-133a-3p.

The purpose of this study was to see if FGFR1 was a downstream effector of lncR-133a/miR-133a-3p that mediated the regulation of MuSC differentiation. FGFR1 overexpression promoted myocyte proliferation and delayed differentiation in mice [49]. Conversely, the expression of mutated FGFR1 enhanced myocyte differentiation [49]. In addition, the knockdown of FGFR1 and PP2AC by siRNA promoted C2C12 differentiation [30]. In line with these studies, our findings suggest that FGFR1 inhibits MuSC differentiation in goats. The interaction between FGFR1 and miR-133a-3p was assessed using a dual-luciferase reporter system. Mutations in the predicted miR-133a-3p target site in the 3′ UTR of the FGFR1 gene abolished luciferase activity, demonstrating that FGFR1 was a target of miR-133a-3p. Moreover, qRT-PCR results indicated that miR-133a-3p inhibited FGFR1 expression during MuSC differentiation. Further experiments revealed that lncR-133a increased FGFR1 expression by sponging miR-133a-3p. Previous study revealed that miR-133 inhibits myoblast proliferation and promotes myoblast differentiation by repressing the ERK1/2 pathway via FGFR1 and PP2AC [30]. Furthermore, ERK1/2 inhibits myoblast differentiation in C2C12 cells [50]. We found that lncR-133a promoted ERK1/2 phosphorylation, suggesting that lncR-133a might regulate MuSC differentiation by activating the ERK1/2 signaling pathway.

## 5. Conclusions

**Figure 7:**
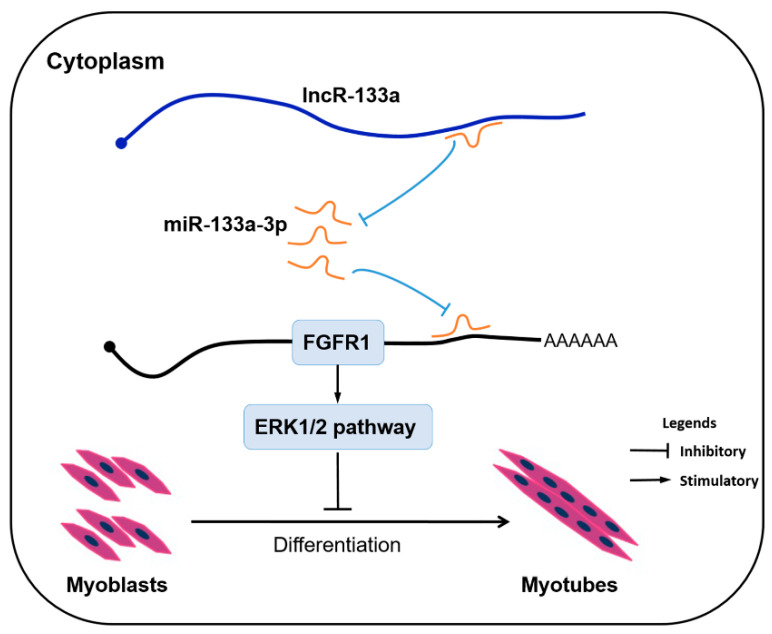
A proposed model of lncR-133a-mediated regulation of myogenic differentiation. LncR-133a activates FGFR1/ERK1/2 signaling and suppresses muscle differentiation by sponging miR-133a-3p.

In conclusion, we identified a novel lncR-133a as a sponge for miR-133a-3p to activate FGFR1/ERK1/2 signaling, thus suppressing muscle differentiation (Figure 7). The lncR-133a/miR-133a-3p/FGFR1/ERK1/2 axis may shed light on further understanding the skeletal muscle development mechanism. Furthermore, our findings suggest that lncR-133a is a potential regulatory element for muscle development and myogenesis in goats.

## Data Availability

The datasets used and/or analyzed during the current study are available from the corresponding author on reasonable request.

## Supplementary Materials

The following supporting information can be downloaded at: https://www.mdpi.com/article/10.3390/genes13050818/s1, Figure S1: The sequence information of goat lncR-133a; Figure S2: Expression pattern of myogenesis marker genes (Pax7, MyoD, MyoG, and MyHC). (**a**) qRT-PCR measured the expression levels of four marker genes in goat different tissues on day 60 of gestation. (**b**) The expression levels of four marker genes in the developmental LTL muscles aged from E45 to B3. (**c**) Real-time PCR analysis of four marker genes expression in MuSCs during 7 days of differentiation. Data are means ± standard error of the mean of at least three biological replicates, * *p* < 0.05; Table S1: Specific primers used in this study.
