# Supplementary material for: LncR-133a Suppresses Myoblast Differentiation by Sponging miR-133a-3p to Activate the FGFR1/ERK1/2 Signaling Pathway in Goats
Source: Genes (Basel). 2022 May 3;13(5):818. doi: 10.3390/genes13050818 (PMC9141198; doi:10.3390/genes13050818)
Supplement: Supplementary file 1 [file genes-13-00818-s001.zip › Table_S1.pdf]

Table S1. Specific primers used in this study.

Name	Sequence of primer (5'→3')	Tm (°C)	Product size(bp)
lncR-133a	F: GGAAAAGCAAGACGAGGGACA	60.7	128
	R: GACAGGGGTAGGGTGGGGGGT		
MyoD	F: GTGCAAACGCAAGACGACTA	60.7	128
	R: GCTGGTTTGGGTTGCTAGAC		
MyoG	F: GGACCCTACAGATGCCCACAA	60.7	101
	R: TTGGTATGGTTTCATCTGGG		
Pax7	F: AGGACGAAGCGGACAAGAA	59.7	92
	R: TCCAGACGGTTCCCTTTGT		
MyHC	F: CCACATCTTCTCCATCTCTG	61.3	171
	R: GGTTCCTCCTTCTTCTTCTC		
miR-133a-3p	F: TTTGGTCCCCTTCAACCAGCTGT	53	—
FGFR1	F: GGCTACAAGGTCCGTTATGC	60.7	101
	R: GATGCTGCCGTATTCGTT		
Actin	F: CCTGCGGCATTCACGAAACTAC	59.7	87
	R: ACAGCACCGTGTTGGCGTAGAG		
U6	F: CAAGGATGACACGCAAATTCG	59	—
18S rRNA	F: GAGAAACGGCTACCACATCC	60	139
	R: GCCAGACTTGCCCTCCA		
pEGFP-lncR-133a	F: CCCAAGCTTCTGTCCACTGCCATCCCCTGCTTCCTT	53	215
	R: CGGGATCCCTCTTCTGCGGCTTCTTTTCCTACCCC		
lncR-133a-wild	F: CCGCTCGAGCCCCTCTATTTCTGTTGGAT	59	215
	R: AAATATGCGGCCGCATTCCTCAAGTTCTACCCTGT		
pEGFP-FGFR1	F:CCGCTCGAGAAATCTTCACTCTGGGCGGCT	60	395
	R:AAATATGCGGCCGCGCGCCGTTTGAGTCCGC		
FGFR1-wild	F: CCGCTCGAGCCCTCCTCTCCTCCTCACAG	60	299
	R: AAATATGCGGCCGCCCATCTGGAACAGAGGTCGC		
