# Supplementary figures and images for: LncR-133a Suppresses Myoblast Differentiation by Sponging miR-133a-3p to Activate the FGFR1/ERK1/2 Signaling Pathway in Goats
Source: Genes (Basel). 2022 May 3;13(5):818. doi: 10.3390/genes13050818 (PMC9141198; doi:10.3390/genes13050818)

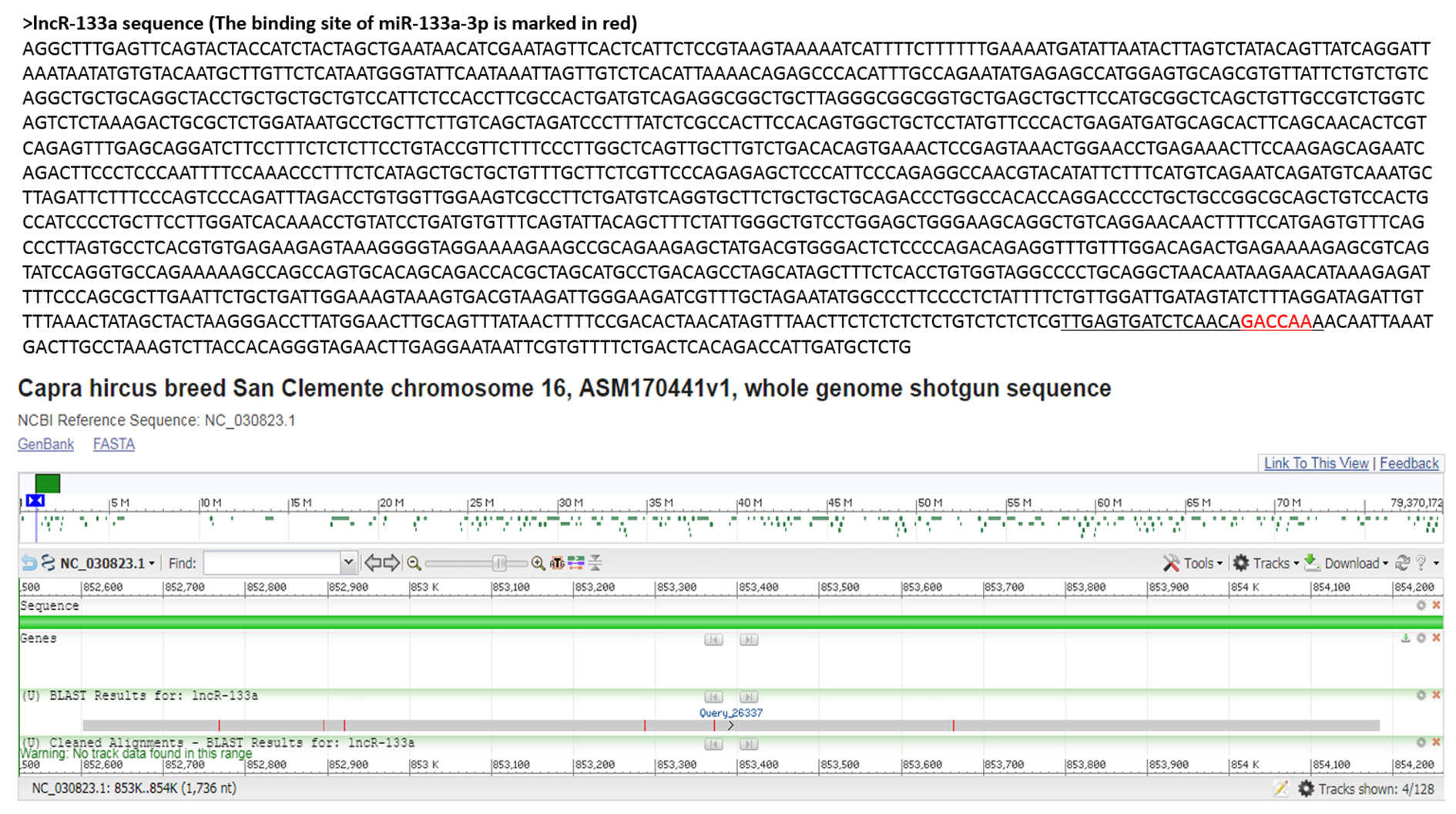

Supplement: Supplementary file 1 [file genes-13-00818-s001.zip › Figure S1.tif]

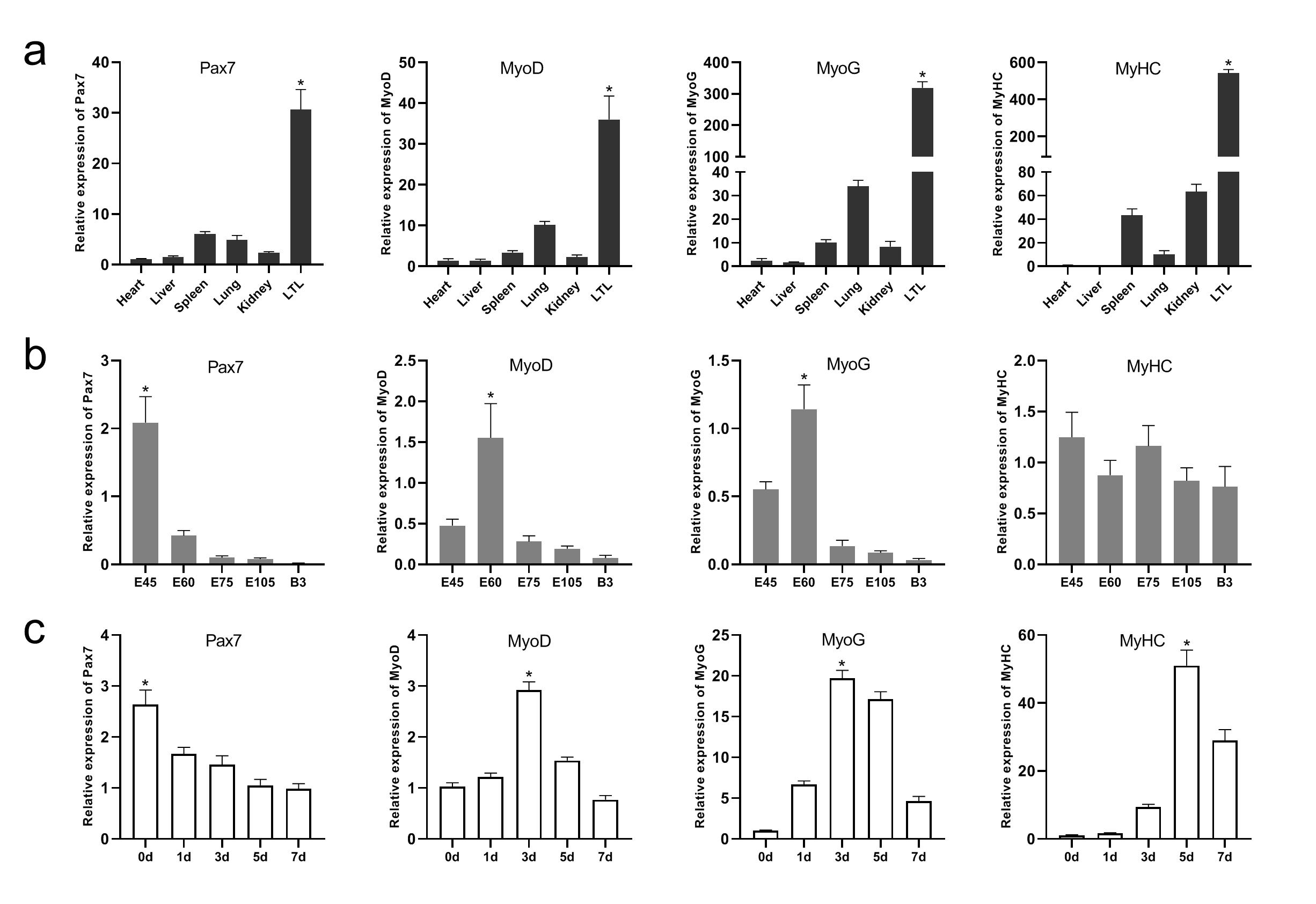

Supplement: Supplementary file 1 [file genes-13-00818-s001.zip › Figure S2.tif]
